# Crystal structure of 2-ethyl­quinazoline-4(3*H*)-thione

**DOI:** 10.1107/S160053681401664X

**Published:** 2014-08-01

**Authors:** Mohammed B. Alshammari, Keith Smith, Amany S. Hegazy, Benson M. Kariuki, Gamal A. El-Hiti

**Affiliations:** a Chemistry Department, College of Sciences and Humanities, Salman bin Abdulaziz University, PO Box 83, Al-Kharij 11942, Saudi Arabia; b School of Chemistry, Cardiff University, Main Building, Park Place, Cardiff CF10 3AT, Wales; c Cornea Research Chair, Department of Optometry, College of Applied Medical Sciences, King Saud University, PO Box 10219, Riyadh 11433, Saudi Arabia

**Keywords:** crystal structure, N—H⋯S inter­actions, quinazoline-4(3*H*)-thione, hydrogen-bonded dimers, herringbone arrangement

## Abstract

In the title compound, C_10_H_10_N_2_S, all non-H atoms are almost coplanar [maximum deviation = 0.103 (1) Å]. In the crystal, N—H⋯S inter­actions form *R*_2_^2^(8) rings linking pairs of mol­ecules related by inversion. The mol­ecular pairs are stacked along [100]. A herringbone arrangement of pairs in the [010] direction forms layers parallel to (010).

## Related literature   

For the synthesis of quinazoline-4(3*H*)-thio­nes, see: Bogert *et al.* (1903 ▶); Zoltewicz & Sharpless (1976 ▶); Segarra *et al.* (1998 ▶); El-Hiti (2004 ▶); Ozturk *et al.* (2007 ▶); El-Hiti *et al.* (2011 ▶).
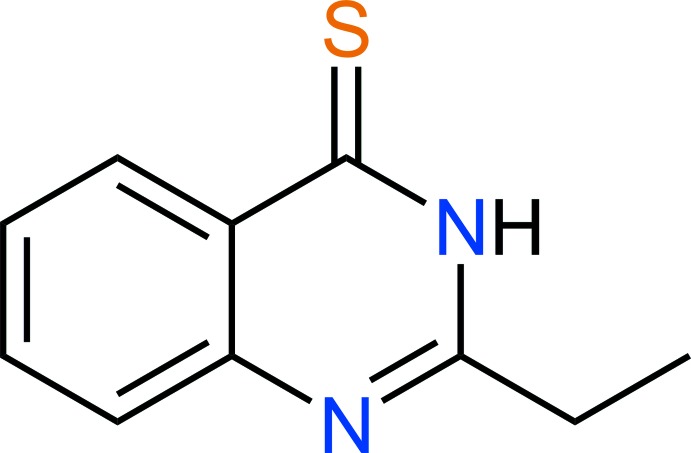


## Experimental   

### Crystal data  

C_10_H_10_N_2_S
*M* *_r_* = 190.26
Orthorhombic, 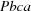

*a* = 5.8231 (3) Å
*b* = 14.3214 (6) Å
*c* = 21.7365 (8) Å
*V* = 1812.71 (14) Å^3^
*Z* = 8
Mo *K*α radiation
μ = 0.31 mm^−1^
*T* = 150 K
0.41 × 0.24 × 0.15 mm

### Data collection  

Agilent SuperNova (Dual, Cu at zero, Atlas) diffractometer
Absorption correction: multi-scan (*CrysAlis PRO*; Agilent, 2014 ▶) *T*_min_ = 0.780, *T*_max_ = 1.000
7795 measured reflections
2240 independent reflections
1973 reflections with *I* > 2σ(*I*)
*R*_int_ = 0.020

### Refinement  

*R*[*F* ^2^ > 2σ(*F* ^2^)] = 0.033
*wR*(*F* ^2^) = 0.087
*S* = 1.03
2240 reflections
119 parameters
H-atom parameters constrained
Δρ_max_ = 0.30 e Å^−3^
Δρ_min_ = −0.25 e Å^−3^

### 

Data collection: *CrysAlis PRO* (Agilent, 2014 ▶); cell refinement: *CrysAlis PRO*; data reduction: *CrysAlis PRO*; program(s) used to solve structure: *SHELXTL* (Sheldrick, 2008 ▶); program(s) used to refine structure: *SHELXTL*; molecular graphics: *SHELXTL*; software used to prepare material for publication: *SHELXTL*.

## Supplementary Material

Crystal structure: contains datablock(s) I, New_Global_Publ_Block. DOI: 10.1107/S160053681401664X/xu5804sup1.cif

Structure factors: contains datablock(s) I. DOI: 10.1107/S160053681401664X/xu5804Isup2.hkl

Supporting information file. DOI: 10.1107/S160053681401664X/xu5804Isup3.cml

**Fig. 1.**
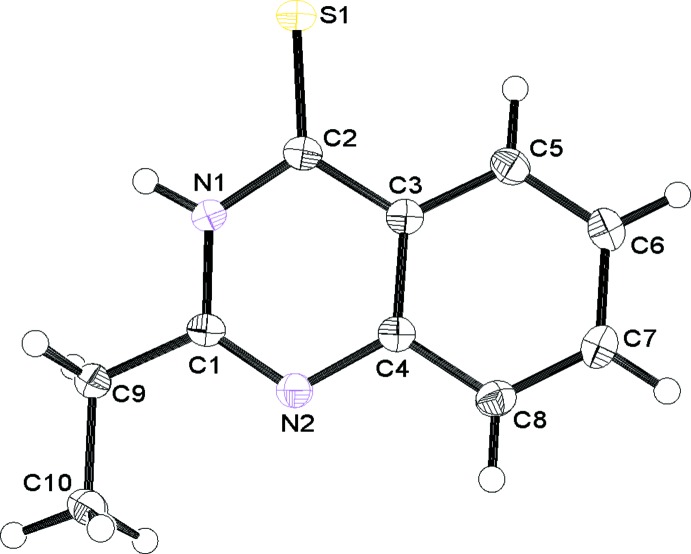
A molecule of the title compound showing atom labels and 50% probability displacement ellipsoids for non-H atoms.

. DOI: 10.1107/S160053681401664X/xu5804fig1.tif
A mol­ecule of the title compound showing atom labels and 50% probability displacement ellipsoids for non-H atoms.

**Fig. 2.**
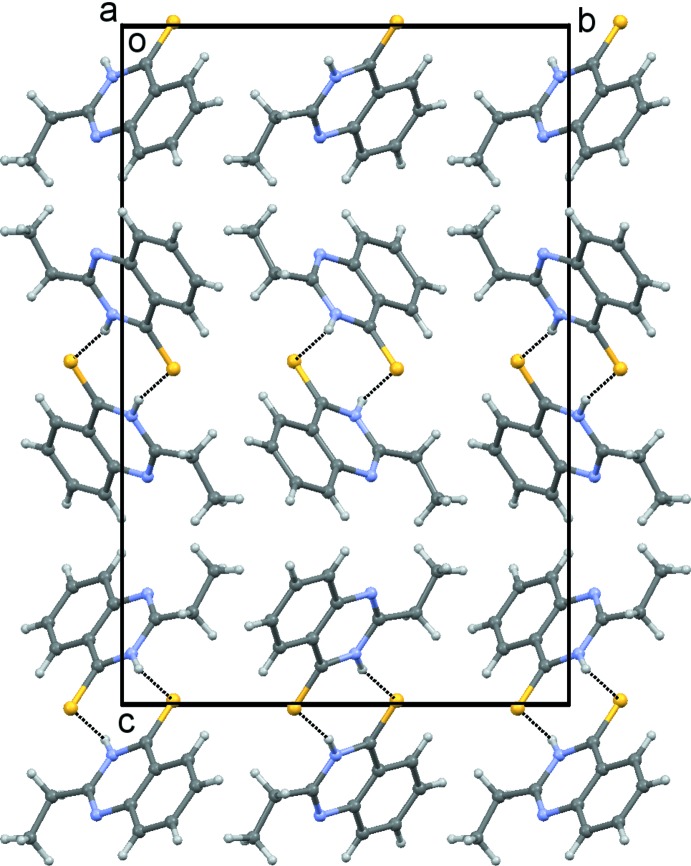
Crystal structure packing showing N—H···S contacts as dotted lines.

. DOI: 10.1107/S160053681401664X/xu5804fig2.tif
Crystal structure packing showing N—H⋯S contacts as dotted lines.

CCDC reference: 1014729

Additional supporting information:  crystallographic information; 3D view; checkCIF report

## Figures and Tables

**Table 1:** Hydrogen-bond geometry (Å, °)

*D*—H⋯*A*	*D*—H	H⋯*A*	*D*⋯*A*	*D*—H⋯*A*
N1—H1⋯S1^i^	0.88	2.53	3.3854 (11)	166

Symmetry code: (i) 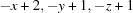.

## supplementary crystallographic information

### S2. Structural commentary

The 2-ethyl-3*H*-quinazoline-4-thione molecule (Fig 1) is almost planer (apart from the ethyl hydrogens) with the ethyl group being twisted from the quinazoline-4-thione plane by 8.7 (2)°. N—H···S inter­actions form R_2_^2^(8) rings to link pairs of molecules related by inversion. The pairs of molecules are stacked parallel to the *a*-axis (Fig 2). Adjacent pairs pack in a herring bone arrangement in the [010] direction to form layers parallel to the (010) plane. 2-(Substituted alkyl)-3*H*-quinazoline-4-thione derivatives can be obtained from double li­thia­tion of 2-alkyl-3*H*-quinazoline-4-thio­nes followed by reactions with electrophiles, including alkyl iodides, at low temperature in anhydrous THF (El-Hiti, 2004). Also, 3*H*-quinazoline-4-thio­nes are produced from the corresponding 3*H*-quinazoline-4-ones using phospho­rus penta­sulfide (Bogert *et al.*, 1903; Ozturk *et al.*, 2007; El-Hiti *et al.*, 2011) or Lawesson's reagent (Segarra *et al.*, 1998). 3*H*-Quinazoline-4-thio­nes have also been synthesized in one-step from reaction of 2-amino­benzo­nitriles and thio­amides in the presence of hydrogen bromide in various solvents on a steam bath for 1–4 h (Zoltewicz & Sharpless, 1976).

### S5. Synthesis and crystallization

2-Ethyl-3*H*-quinazoline-4-thione was obtained in 92% yield from double li­thia­tion of 2-methyl-3*H*-quinazoline-4-thione with *n*-butyl­lithium at 78 ^ο^C in anhydrous THF under nitro­gen followed by reaction with iodo­methane (El-Hiti, 2004). Crystallization from methanol gave the title compound as yellow crystals. The NMR and low and high resolution mass spectra for the title compound were consistent with those reported (El-Hiti, 2004).

### S6. Refinement

Crystal data, data collection and structure refinement details are summarized in Table 1. H atoms were placed in calculated positions with C—H = 0.95 and 0.98Å and refined in riding mode, *U*_iso_(H) = 1.5*U*_eq_(C) for methyl H atoms and 1.2Ueq(C) for aromatic H atoms

### Crystal data

**Table:**

C_10_H_10_N_2_S	*D*_x_ = 1.394 Mg m^−^^3^
*M**_r_* = 190.26	Mo *K*α radiation, λ = 0.71073 Å
Orthorhombic, *P**b**c**a*	Cell parameters from 3617 reflections
*a* = 5.8231 (3) Å	θ = 3.9–29.3°
*b* = 14.3214 (6) Å	µ = 0.31 mm^−^^1^
*c* = 21.7365 (8) Å	*T* = 150 K
*V* = 1812.71 (14) Å^3^	Plate, yellow
*Z* = 8	0.41 × 0.24 × 0.15 mm
*F*(000) = 800	

### Data collection

**Table:**

Agilent SuperNova (Dual, Cu at zero, Atlas) diffractometer	2240 independent reflections
Radiation source: SuperNova (Mo) X-ray Source	1973 reflections with *I* > 2σ(*I*)
Mirror monochromator	*R*_int_ = 0.020
ω scans	θ_max_ = 29.8°, θ_min_ = 3.0°
Absorption correction: multi-scan (*CrysAlis PRO*; Agilent, 2014)	*h* = −6→7
*T*_min_ = 0.780, *T*_max_ = 1.000	*k* = −19→14
7795 measured reflections	*l* = −23→29

### Refinement

**Table:**

Refinement on *F*^2^	0 restraints
Least-squares matrix: full	Hydrogen site location: inferred from neighbouring sites
*R*[*F*^2^ > 2σ(*F*^2^)] = 0.033	H-atom parameters constrained
*wR*(*F*^2^) = 0.087	*w* = 1/[σ^2^(*F*_o_^2^) + (0.0416*P*)^2^ + 0.7981*P*] where *P* = (*F*_o_^2^ + 2*F*_c_^2^)/3
*S* = 1.03	(Δ/σ)_max_ < 0.001
2240 reflections	Δρ_max_ = 0.30 e Å^−^^3^
119 parameters	Δρ_min_ = −0.25 e Å^−^^3^

### Special details

**Table:**

Geometry. All e.s.d.'s (except the e.s.d. in the dihedral angle between two l.s. planes) are estimated using the full covariance matrix. The cell e.s.d.'s are taken into account individually in the estimation of e.s.d.'s in distances, angles and torsion angles; correlations between e.s.d.'s in cell parameters are only used when they are defined by crystal symmetry. An approximate (isotropic) treatment of cell e.s.d.'s is used for estimating e.s.d.'s involving l.s. planes.

### Fractional atomic coordinates and isotropic or equivalent isotropic displacement parameters (Å^2^)

**Table:**

	*x*	*y*	*z*	*U*_iso_*/*U*_eq_	
C1	0.7433 (2)	0.57478 (9)	0.62537 (6)	0.0185 (3)	
C2	0.6649 (2)	0.44643 (8)	0.55604 (5)	0.0178 (2)	
C3	0.4705 (2)	0.42557 (8)	0.59550 (5)	0.0177 (3)	
C4	0.4368 (2)	0.48131 (8)	0.64832 (5)	0.0183 (3)	
C5	0.3171 (2)	0.35262 (9)	0.58239 (6)	0.0209 (3)	
H5	0.3379	0.3158	0.5465	0.025*	
C6	0.1366 (2)	0.33425 (9)	0.62146 (6)	0.0236 (3)	
H6	0.0337	0.2845	0.6127	0.028*	
C7	0.1046 (2)	0.38917 (9)	0.67440 (6)	0.0240 (3)	
H7	−0.0197	0.3760	0.7013	0.029*	
C8	0.2513 (2)	0.46175 (9)	0.68761 (6)	0.0218 (3)	
H8	0.2272	0.4987	0.7233	0.026*	
C9	0.8998 (2)	0.65696 (9)	0.63452 (6)	0.0227 (3)	
H9A	0.8734	0.7023	0.6009	0.027*	
H9B	1.0609	0.6354	0.6316	0.027*	
C10	0.8668 (3)	0.70632 (9)	0.69567 (6)	0.0246 (3)	
H10A	0.7058	0.7252	0.7000	0.037*	
H10B	0.9652	0.7618	0.6972	0.037*	
H10C	0.9080	0.6639	0.7293	0.037*	
N1	0.78973 (18)	0.52156 (7)	0.57402 (5)	0.0188 (2)	
H1	0.9086	0.5374	0.5513	0.023*	
N2	0.57662 (19)	0.55697 (7)	0.66273 (5)	0.0199 (2)	
S1	0.73978 (6)	0.38502 (2)	0.49367 (2)	0.02214 (11)	

### Atomic displacement parameters (Å^2^)

**Table:**

	*U*^11^	*U*^22^	*U*^33^	*U*^12^	*U*^13^	*U*^23^
C1	0.0210 (6)	0.0177 (6)	0.0169 (6)	0.0022 (5)	−0.0006 (4)	−0.0010 (5)
C2	0.0203 (6)	0.0164 (5)	0.0166 (5)	0.0039 (5)	−0.0026 (5)	0.0008 (4)
C3	0.0194 (6)	0.0171 (6)	0.0167 (5)	0.0027 (5)	−0.0019 (5)	0.0020 (4)
C4	0.0197 (6)	0.0178 (6)	0.0172 (5)	0.0019 (5)	−0.0012 (4)	0.0010 (5)
C5	0.0237 (7)	0.0189 (6)	0.0202 (6)	0.0008 (5)	−0.0032 (5)	−0.0006 (5)
C6	0.0238 (7)	0.0212 (6)	0.0258 (6)	−0.0033 (5)	−0.0029 (5)	0.0012 (5)
C7	0.0213 (7)	0.0264 (7)	0.0244 (6)	−0.0013 (5)	0.0028 (5)	0.0038 (5)
C8	0.0242 (7)	0.0226 (6)	0.0186 (6)	0.0015 (5)	0.0016 (5)	0.0002 (5)
C9	0.0238 (7)	0.0208 (6)	0.0233 (6)	−0.0033 (5)	0.0041 (5)	−0.0040 (5)
C10	0.0292 (7)	0.0233 (6)	0.0214 (6)	−0.0047 (5)	0.0001 (5)	−0.0040 (5)
N1	0.0188 (5)	0.0194 (5)	0.0182 (5)	−0.0003 (4)	0.0030 (4)	−0.0024 (4)
N2	0.0220 (6)	0.0191 (5)	0.0187 (5)	−0.0006 (4)	0.0015 (4)	−0.0012 (4)
S1	0.0244 (2)	0.02191 (18)	0.02011 (17)	0.00029 (12)	0.00293 (12)	−0.00599 (12)

### Geometric parameters (Å, º)

**Table:**

C1—N2	1.2908 (16)	C6—C7	1.4061 (19)
C1—N1	1.3784 (16)	C6—H6	0.9500
C1—C9	1.5017 (18)	C7—C8	1.3757 (19)
C2—N1	1.3560 (16)	C7—H7	0.9500
C2—C3	1.4514 (17)	C8—H8	0.9500
C2—S1	1.6737 (12)	C9—C10	1.5177 (17)
C3—C5	1.4038 (18)	C9—H9A	0.9900
C3—C4	1.4119 (16)	C9—H9B	0.9900
C4—N2	1.3910 (16)	C10—H10A	0.9800
C4—C8	1.4054 (18)	C10—H10B	0.9800
C5—C6	1.3766 (19)	C10—H10C	0.9800
C5—H5	0.9500	N1—H1	0.8800
			
N2—C1—N1	123.21 (12)	C6—C7—H7	119.6
N2—C1—C9	121.86 (11)	C7—C8—C4	120.08 (12)
N1—C1—C9	114.92 (11)	C7—C8—H8	120.0
N1—C2—C3	114.27 (11)	C4—C8—H8	120.0
N1—C2—S1	120.71 (10)	C1—C9—C10	113.84 (11)
C3—C2—S1	125.01 (10)	C1—C9—H9A	108.8
C5—C3—C4	119.83 (12)	C10—C9—H9A	108.8
C5—C3—C2	121.97 (11)	C1—C9—H9B	108.8
C4—C3—C2	118.19 (11)	C10—C9—H9B	108.8
N2—C4—C8	117.93 (11)	H9A—C9—H9B	107.7
N2—C4—C3	122.83 (11)	C9—C10—H10A	109.5
C8—C4—C3	119.22 (12)	C9—C10—H10B	109.5
C6—C5—C3	120.19 (12)	H10A—C10—H10B	109.5
C6—C5—H5	119.9	C9—C10—H10C	109.5
C3—C5—H5	119.9	H10A—C10—H10C	109.5
C5—C6—C7	119.94 (12)	H10B—C10—H10C	109.5
C5—C6—H6	120.0	C2—N1—C1	124.53 (11)
C7—C6—H6	120.0	C2—N1—H1	117.7
C8—C7—C6	120.73 (13)	C1—N1—H1	117.7
C8—C7—H7	119.6	C1—N2—C4	116.89 (11)

### Hydrogen-bond geometry (Å, º)

**Table:**

*D*—H···*A*	*D*—H	H···*A*	*D*···*A*	*D*—H···*A*
N1—H1···S1^i^	0.88	2.53	3.3854 (11)	166

Symmetry code: (i) −*x*+2, −*y*+1, −*z*+1.

## References

[bb1] Agilent (2014). *CrysAlis PRO* Agilent Technologies, Yarnton, England.

[bb2] Bogert, M. T., Breneman, H. C. & Hand, W. F. (1903). *J. Am. Chem. Soc.* **25**, 372–380.

[bb3] El-Hiti, G. A. (2004). *Synthesis*, pp. 363–368.

[bb4] El-Hiti, G. A., Hussain, A., Hegazy, A. S. & Alotaibi, M. H. (2011). *J. Sulfur Chem.* **32**, 361–395.

[bb5] Ozturk, T., Ertas, E. & Mert, O. (2007). *Chem. Rev.* **107**, 5210–5278. 10.1021/cr040650b 17867708

[bb6] Segarra, V., Crespo, M. I., Pujol, F., Beleta, J., Doménech, T., Miralpeix, M., Palacios, J. M., Castro, A. & Martinez, A. (1998). *Bioorg. Med. Chem. Lett.* **8**, 505–510. 10.1016/s0960-894x(98)00058-4 9871607

[bb7] Sheldrick, G. M. (2008). *Acta Cryst.* A**64**, 112–122. 10.1107/S0108767307043930 18156677

[bb8] Zoltewicz, J. A. & Sharpless, T. W. (1976). *J. Org. Chem.* **32**, 2681–2685.

