# Supplementary figures and images for: Crystal structure of 2-ethyl­quinazoline-4(3H)-thione
Source: Acta Crystallogr Sect E Struct Rep Online. 2014 Aug 1;70(Pt 9):o953. doi: 10.1107/S160053681401664X (PMC4186129; doi:10.1107/S160053681401664X)

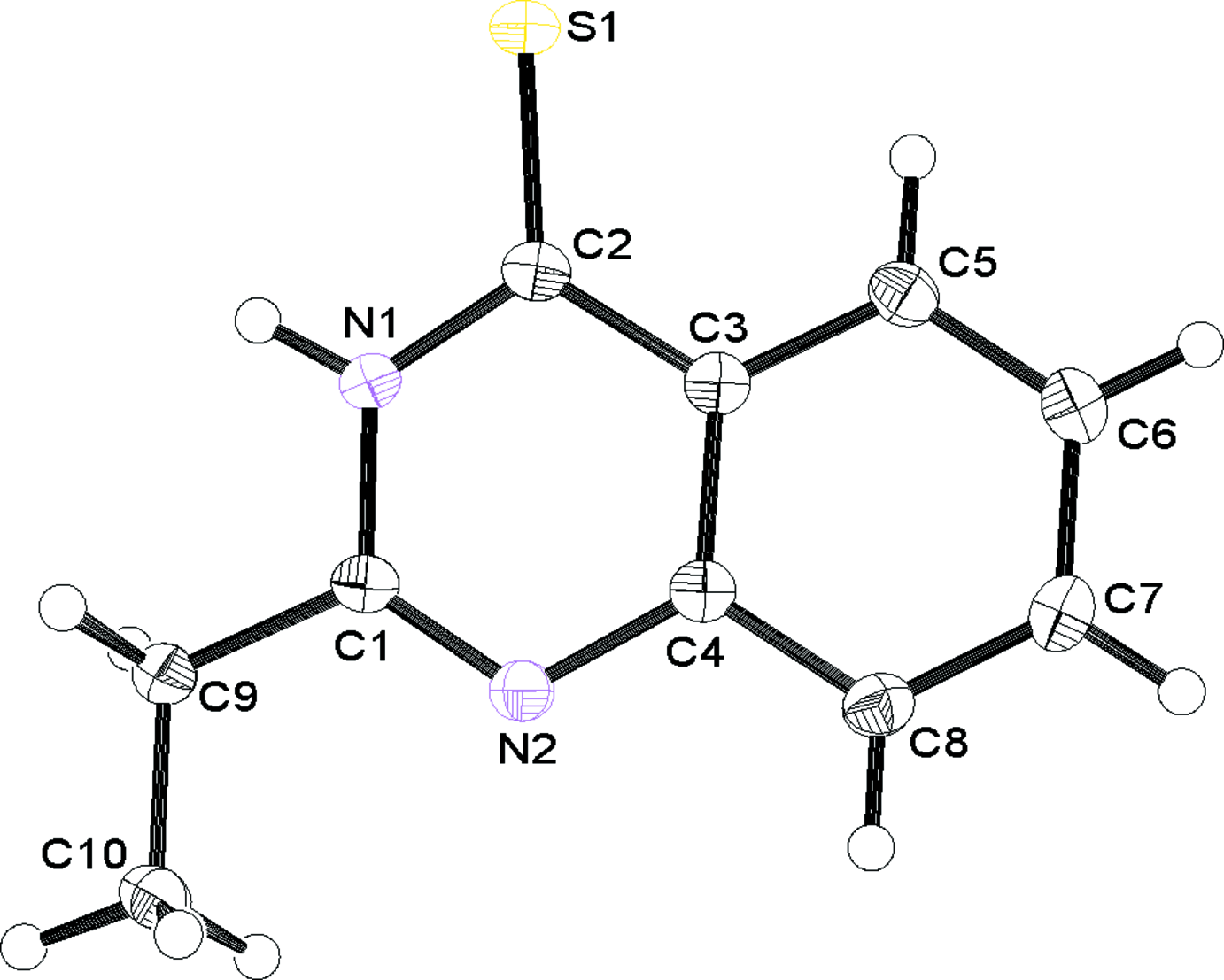

Supplement: Supplementary file 4 [file e-70-0o953-fig1.tif]

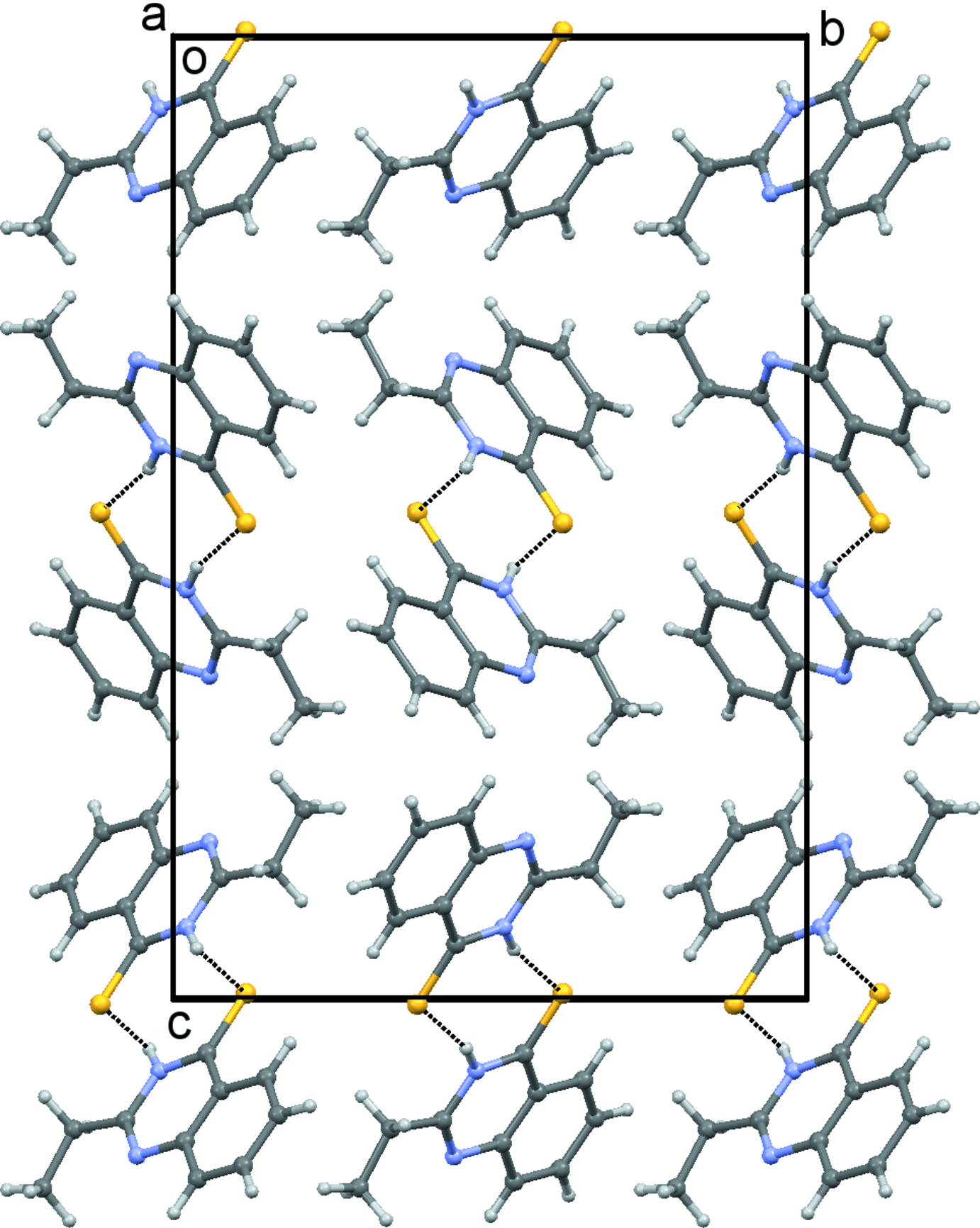

Supplement: Supplementary file 5 [file e-70-0o953-fig2.tif]
